# Sensitization of tumour cells to lysis by virus-specific CTL using antibody-targeted MHC class I/peptide complexes

**DOI:** 10.1054/bjoc.1999.1042

**Published:** 2000-02-01

**Authors:** G S Ogg, P R Dunbar, V Cerundolo, A J McMichael, N R Lemoine, P Savage

**Affiliations:** MRC Human Immunology Unit, Institute of Molecular Medicine, Oxford, OX3 9D5, UK; ICRF Molecular Oncology Unit, Imperial College School of Medicine, London, 12 0HS, UK; Department of Oncology, Velindre Hospital, Velindre Road, Whitchurch, Cardiff, CF4 7XL, UK

**Keywords:** cancer, immunotherapy, monoclonal antibody, HLA class I

## Abstract

A number of cell surface molecules with specificity to tumour cells have been identified and monoclonal antibodies (mAb) to some of these antigens have been used for targeting tumour cells in vivo. We have sought to link the powerful effector mechanisms of cytotoxic T-cells with the specificity of mAb, by targeting recombinant HLA class I molecules to tumour cells using an antibody delivery system. Soluble recombinant MHC class I/peptide complexes including HLA-A2.1 refolded around an immunodominant peptide from the HIV gag protein (HLA-A2/gag) were synthesized, and the stability of these complexes at 37°C was confirmed by enzyme-linked immunosorbent assay using a conformation-specific antibody. MHC class I-negative lymphoma cells (Daudi) were labelled with a biotinylated mAb specific for a cell surface protein (anti-CD20) then linked to soluble biotinylated HLA-A2/gag complexes using an avidin bridge. Flow cytometry revealed strong labelling of lymphoma cells with HLA-A2/gag complexes (80-fold increase in mean channel fluorescence). CTL specific for HLA-A2/gag efficiently lysed complex-targeted cells, while control CTL (specific for an HLA-A2.1-restricted epitope of melan-A) did not. Similarly, SK-mel-29 melanoma cells were also efficiently lysed by HLA-A2/gag-specific CTL when HLA-A2/gag complexes were linked to their surface via the HMW-MAA specific anti-melanoma antibody 225.28s. With further consideration to the in vivo stability of the MHC class I/peptide complexes, this system could prove a new strategy for the immunological therapy of cancer. © 2000 Cancer Research Campaign


British Journal of Cancer (2000) 82(5), 1058–1062
© 2000 Cancer Research Campaign
DOI: 10.1054/ bjoc.1999.1042, available online at http://www.idealibrary.com on IDEAL®

### Sensitization of tumour cells to lysis by virus-specific CTL using antibody-targeted MHC class I/peptide complexes

**GS Ogg[1], PR Dunbar[1], V Cerundolo[1], AJ McMichael[1], NR Lemoine[2] and P Savage[3]**

[1]MRC Human Immunology Unit, Institute of Molecular Medicine, Oxford OX3 9D5, UK; [2]ICRF Molecular Oncology Unit, Imperial College School of Medicine, London W12 0HS, UK; [3]Department of Oncology, Velindre Hospital, Velindre Road, Whitchurch, Cardiff CF4 7XL, UK

**Summary** A number of cell surface molecules with specificity to tumour cells have been identified and monoclonal antibodies (mAb) to some of these antigens have been used for targeting tumour cells in vivo. We have sought to link the powerful effector mechanisms of cytotoxic T-cells with the specificity of mAb, by targeting recombinant HLA class I molecules to tumour cells using an antibody delivery system. Soluble recombinant MHC class I/peptide complexes including HLA-A2.1 refolded around an immunodominant peptide from the HIV gag protein (HLA-A2/gag) were synthesized, and the stability of these complexes at 37°C was confirmed by enzyme-linked immunosorbent assay using a conformation-specific antibody. MHC class I-negative lymphoma cells (Daudi) were labelled with a biotinylated mAb specific for a cell surface protein (anti-CD20) then linked to soluble biotinylated HLA-A2/gag complexes using an avidin bridge. Flow cytometry revealed strong labelling of lymphoma cells with HLA-A2/gag complexes (80-fold increase in mean channel fluorescence). CTL specific for HLA-A2/gag efficiently lysed complex-targeted cells, while control CTL (specific for an HLA-A2.1-restricted epitope of melan-A) did not. Similarly, SK-mel-29 melanoma cells were also efficiently lysed by HLA-A2/gag-specific CTL when HLA-A2/gag complexes were linked to their surface via the HMW-MAA specific anti-melanoma antibody 225.28s. With further consideration to the in vivo stability of the MHC class I/peptide complexes, this system could prove a new strategy for the immunological therapy of cancer. © 2000 Cancer Research Campaign

**Keywords:** cancer; immunotherapy; monoclonal antibody; HLA class I

Considerable evidence points to the efficacy of CTL in clearing viral infections, via the specific recognition of immunogenic viral peptides in the binding groove of the MHC class I molecules (Townsend et al, 1989). However, the efficacy of CTL responses against tumours may be limited by a number of tumour escape mechanisms (Bodmer et al, 1993). Many cancer cells express tumour-associated antigens (TAAs) that can be bound on their surface by monoclonal antibodies (mAbs) (Riethmuller and Johnson 1992). The clinical use of mAbs as native proteins or to direct radioactivity or toxic drugs to tumour cells has been investigated extensively over the past 20 years and now antibodies to treat lymphoma (Maloney et al, 1994), colorectal cancer (Riethmuller et al, 1998) and ovarian cancer (Hird et al, 1993) are entering clinical practice.

Whilst the natural antibody effector mechanisms such as complement-mediated lysis and antibody-dependent cell-mediated cytotoxicity (ADCC) may produce target cell damage in vivo, an alternative of using antibodies to redirect the cellular immune system to tumours may produce a more effective action. A number of immunotherapeutic strategies have been described that combine the tumour specificity of anti-tumour mAbs with these powerful effector mechanisms of the cellular immune system. Bispecific antibodies which cross-link tumours to receptors on T-cells (Perez et al, 1985), natural killer (NK) cells (Weiner et al, 1996), macrophages and neutrophils (Valone et al, 1995), and the antibody superantigen conjugates (Dohlsten et al, 1991) and fusion proteins (Dohlsten et al, 1993) that link TAAs to T-cell receptors all aim to redirect effector cells of non-tumour specificity to tumour cells.

Here we describe a novel targeting system to deliver MHC class I/peptide complexes to tumour cells via tumour-specific mAb, thereby rendering the tumour cells susceptible to lysis by CTLs which have specificity for the peptide incorporated into the recombinant MHC class I/peptide complex. The result of this will be to allow efficient lysis of tumour cells of low immunogenicity by CTLs of a non-tumour specificity such as anti-viral CTLs (Figure 1).

### MATERIALS AND METHODS

### Cell lines

The Daudi B-cell line (Klein et al, 1968) (MHC class I-negative) melanoma line SK-mel-29 (Knuth et al, 1989) (HLA-A2.1-positive), and 221/A2, an HLA-A2.1-positive T-cell clone, were maintained in RPMI media with 10% fetal calf serum and antibiotics in a 37°C incubator with 5% carbon dioxide. Human cytotoxic T-cell clones 010 (specific for HLA-A2/gag 77–85 = SLYNTVATL) (Parker et al, 1992) and 1F9 (specific for HLA-A2/melan-A 26–35 = EAAGIGILTV) (Romero et al, 1997) were maintained in media supplemented with 5% human serum and IL-2 100 IU $ml^{-1}$.

*Received 4 August 1999*
*Revised 9 October 1999*
*Accepted 12 October 1999*

*Correspondence to:* P Savage

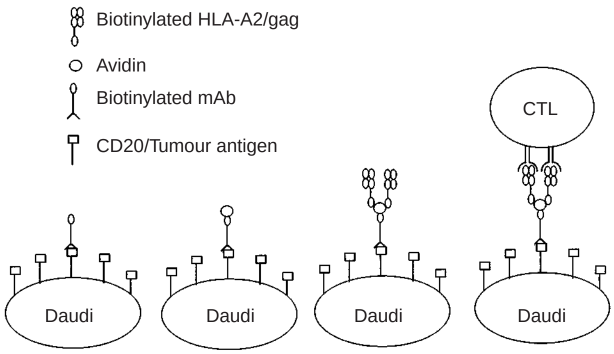

**Figure 1** Schematic representation of the three-step targeting system delivering HLA-A2 peptide complexes to cells bearing a tumour associated antigen. Step 1 is the delivery of a biotinylated mAb with specificity for the tumour associated antigen. Step 2 is the delivery of avidin. Step 3 is the binding of recombinant biotinylated HLA-A2 containing the immunogenic peptide of viral origin

### Production of MHC class I/peptide complexes

Biotinylated complexes of recombinant MHC class I and peptide were produced as described previously (Altman et al, 1996; Ogg et al, 1998). Briefly, prokaryotic expression of $B_2M$ and MHC class I heavy chain, modified by the C terminal addition of a target sequence for the biotin ligase enzyme BirA, was followed by inclusion body purification. Following refolding of heavy chain and $B_2M$ around specific peptide, complexes of 45 kDa were isolated by gel filtration, biotinylated overnight to an efficiency of 70–100% at a single lysine residue within the target sequence peptide using BirA in the presence of ATP, $Mg^{2+}$ and biotin, and then purified by gel filtration and anion exchange.

### Stability of MHC class I/peptide complexes

Complexes at 10 μg $ml^{-1}$ in tissue culture media were preincubated for 0–20 h at 37°C, before analysis by enzyme-linked immunosorbent assay (ELISA). ELISA plates had been coated with the mAb W6/32 (5 μg $ml^{-1}$ in carbonate buffer pH 9.6 overnight at 4°C) that recognizes conformationally-correct MHC class I molecules (Parham et al, 1979), then blocked (1% bovine serum albumin in phosphate-buffered saline (PBS) for 2 h at 37°C). MHC class I complexes were incubated for 30 min at room temperature, and binding detected with rabbit anti-human $\beta_2M$ followed by alkaline phosphatase-conjugated goat anti-rabbit immunoglobulin. Detection of the bound enzyme was by incubation with pNPP with the absorbance at 405 nm measured in a Titertek Multiscan ELISA reader. The plots show the mean of assays performed in triplicate. All incubations were separated by extensive washes in PBS.

### FACS analysis

Daudi cells deficient in MHC class I expression were sequentially incubated at 4°C with biotinylated anti-CD20 (Ancell, Nottingham, UK; mAb 2H7 (Berenson et al, 1986) 1 μg $ml^{-1}$, 30 min), hen egg avidin (S.P.A., Milan, Italy; 10 μg $ml^{-1}$, 10 min), biotinylated HLA-A2/gag (10 μg $ml^{-1}$, 10 min) and fluorescein isothiocyanate (FITC)-labelled anti-MHC class I (Ancell, Nottingham, UK; mAB 3F10 (Eisenbarth et al, 1980) 10 μg $ml^{-1}$). Parallel controls omitted one or other incubation. Cells were washed 3 times in PBS between stages and then fixed in PBS plus 2% formaldehyde and analysed by flow cytometry.

### Cytotoxicity assays

Daudi or SK-mel-29 cells were incubated with $^{51}CrO_4$ at 2 μCi $\mu l^{-1}$ for 1 h at 37°C and then sequentially incubated with the biotinylated mAbs 2H7 or 225.28s (anti-HMW-MAA) (Buraggi et al, 1985) respectively, avidin and biotinylated HLA-A2/gag complexes as detailed above for FACS analysis. Peptide pulsed targets cells were incubated with gag 77–85 or melan-A 26–35 peptides 0.1 μM for 1 h at 37°C. After washing, labelled target cells were plated into 96-well round bottom plates at 2500 cells per well, followed by human CTL at various effector:target ratios. Following incubation at 37°C, 20 μl of supernatant was collected and the amount of $^{51}Cr$ released determined. The percentage of cytotoxicity obtained at each effector:target ratio was calculated as $100 \times (E-M)/(T-M)$, where E = experimental release, M = release in media and T = release in 5% Triton X-100 detergent. Results shown are the mean of experiments performed in duplicate.

### RESULTS

### Biotinylated recombinant MHC class I/peptide complexes are stable at 37°C

The effect on the stability of the recombinant MHC class I/peptide complexes by preincubation at 37°C was demonstrated by ELISA. The optical density obtained with samples preincubated for 0, 1, 4, 16 and 20 h is shown in Figure 2. The results demonstrate that the HLA-A2/gag complexes have appreciable stability in culture media at 37°C, with an estimated half-life in excess of 24 h. Similar results are shown for a number of other MHC class I peptide complexes including HLA-A2/Gag3F(SLFNTVATL), HLA-A2/Lmp2, HLA-B35/Env and HLA-B35/nef. In storage at 0.5–1 mg $ml^{-1}$ at 4°C HLA-A2/gag complexes appear to be stable for at least 12 months (data not shown).

### Targeting of biotinylated HLA-A2/gag complexes to Daudi cells demonstrated by FACS

Cells incubated with all three layers of the labelling system had high levels of detectable MHC class I/peptide on their surface compared to untreated Daudi cells (Figure 3). Cells treated with only any two components of the three-step system gave fluorescence levels comparable to untreated cells (data not shown).

### Tumour cells targeted by HLA-A2/gag complexes are lysed by HLA-A2/gag-specific CTL

CTL clone 010 efficiently lysed HLA-A2-positive targets (221/A2) only when these were preincubated with the HLA-A2/gag peptide (Figure 4A). MHC class I-negative Daudi cells, targeted with HLA-A2/gag complexes, were recognized and lysed by this CTL clone to an equivalent degree (Figure 4A). Untargeted Daudi cells and cells targeted with only two of the three incubations were not recognized (maximal lysis < 4% at E:T ratios of up to 80:1). Control CTL, showing a different HLA-A2-restricted specificity (HLA-A2/melan-A), did not lyse Daudi cells targeted

© 2000 Cancer Research Campaign

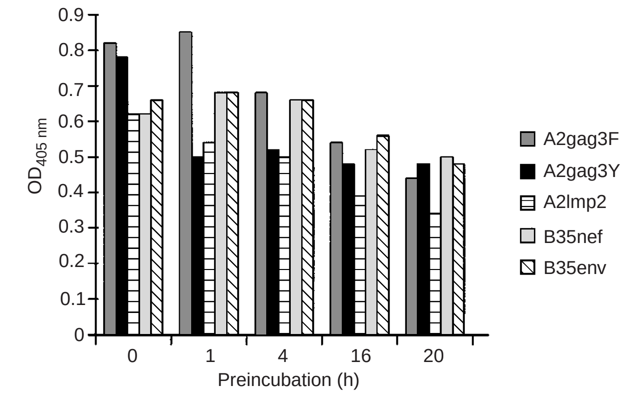

**Figure 2** ELISA result examining the in vitro stability of HLA-A2/peptides complexes following preincubation at 37°C. The HLA is bound by immobilized mAb W6/32 which only recognizes conformationally correct HLA class I and is detected by rabbit anti-human β2-microglobulin

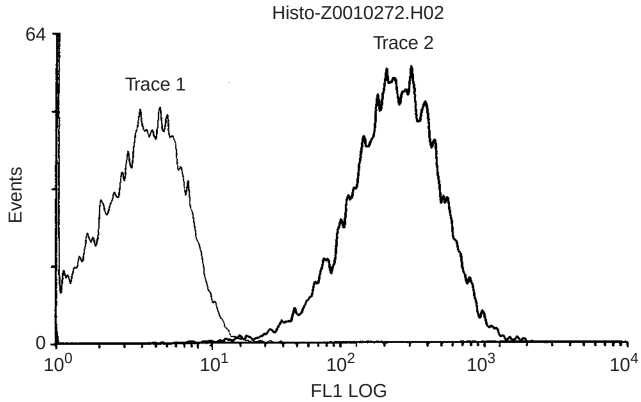

**Figure 3** FACS analysis of MHC class I-deficient Daudi cells targeted with HLA-A2 via biotinylated anti-CD20 mAb. The presence of bound HLA class I molecules was demonstrated with an anti-HLA class I FITC-conjugated mAb. Trace 1 native untreated Daudi cells. Trace 2 Daudi cells targeted sequentially with mAb/avidin/HLA-A2/gag/ followed by FITC-conjugated anti-HLA class I

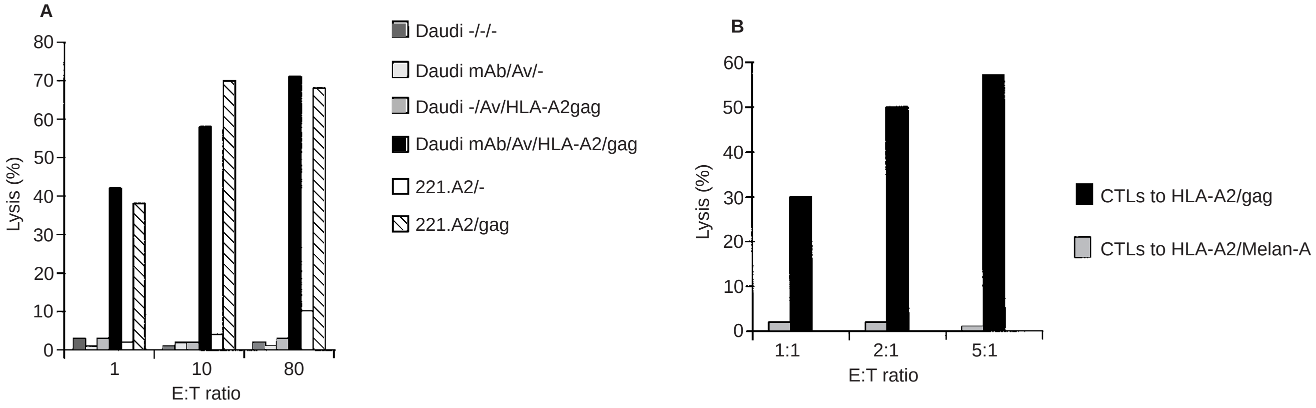

**Figure 4** **A** Four-hour $^{51}$Cr release assay using the HLA-A2/gag-specific CTL clone 010 and HLA-class I-deficient Daudi cell targets targeted with the three-step delivery system. Column 1 is native Daudi cells; column 2 is Daudi cells targeted with steps 1 and 2 only; column 3 is Daudi cells targeted with steps 2 and 3 only; column 4 is Daudi cells targeted with all 3 steps of the system; column 5 is HLA-A2-positive target cells unpulsed; column 6 is HLA-A2-positive target cells pulsed with the gag peptide. The results of duplicate experiments at effector to target ratios of 1:1, 10:1 and 80:1 are displayed. **B** Four-hour $^{51}$Cr release assay using HLA-A2/gag and HLA-A2/Melan A specific CTLs against Daudi cells targeted with HLA-A2/gag complexes using the three-step system

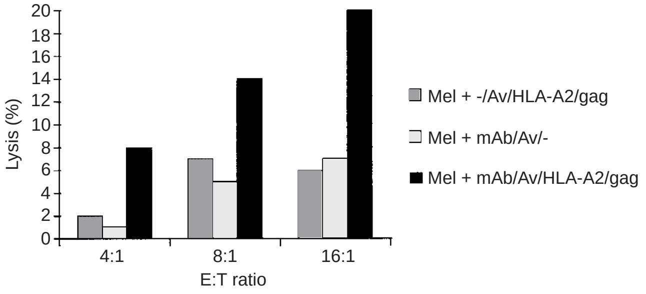

**Figure 5** Twenty-hour $^{51}$Cr release assay using the HLA-A2/gag-specific CTL line and HLA-A2-positive SK-mel-29 cells targeted with two or all three steps of the three-step targeting system. Column 1 is melanoma cells targeted with steps 2 and 3 only; column 2 is melanoma cells targeted with steps 1 and 2 only; column 3 is melanoma cells targeted with all 3 steps. The results of duplicate experiments at effector to target ratios of 4:1, 8:1 and 16:1 are displayed with the HLA-A2/gag complexes (Figure 4B), demonstrating the fine specificity of the targeting approach. Untreated Daudi cells pulsed with gag peptide alone were not lysed by clone 010 (data not shown), in keeping with their lack of endogenous MHC class I.

The ability of antibody-directed HLA-A2/gag complexes to sensitize the melanoma cell line SK-mel-29 to lysis by HLA-A2/gag-specific CTL line in shown in Figure 5. At all E:T ratios, melanoma cells targeted by complexes linked to surface proteins were lysed substantially more than controls exposed to only two components of the three-step targeting system.

### DISCUSSION

Cancer immunotherapy aimed at stimulating tumour-specific CTL has met only limited success to date. A number of alternative approaches using the specificity of antibody–TAA interactions to redirect cytotoxic T-cells of non-tumour specificity to cancer cells are currently in clinical trials. In this project we have investigated the possibility of targeting cancer cells for lysis by virus-specific

 © 2000 Cancer Research Campaign

CTL using soluble MHC class I/peptide complexes. The availability of recombinant HLA class I molecules containing peptides of predetermined specificity (Garboczi et al, 1992) and that can incorporate a biotin binding domain (Altman et al, 1996) has facilitated the investigation of this new therapeutic strategy.

The hypothesis that T-cells will interact effectively with HLA class I molecules attached to cells by an antibody bridge is supported by previously published work. CTL can degranulate and release cytokines on binding immobilized MHC class I/peptide complexes (Kane et al, 1989) and streptavidin-conjugated HLA-A2 attached to murine plasmacytoma cells via biotinylated cell surface proteins can induce effective lysis of these cells by HLA-A2-restricted CTL (Elliot and Eisen, 1988).

The viability of attaching recombinant MHC class I/peptide complexes to tumour cell surface proteins was confirmed by FACS analysis (Figure 3). The ability of CTL to recognize these complexes and specifically lyse the targeted tumour cells was confirmed by chromium release assay (Figures 4 and 5). Lysis of targeted (MHC class I-negative) Daudi cells was extremely efficient, comparable to MHC class I-positive targets (Figure 4A). Hence recombinant soluble MHC classI/peptide complexes remain fully functional when bound to tumour cells in this way.

The results from the lysis of the HLA-A2 expressing melanoma cell line SK-mel-29 indicate that there can still be effective targeting and interaction with T-cells in the presence of endogenous class I on the surface of the target cell. The degree of cell killing seen in this experiment is lower than in the Daudi cell line (Figure 5 cf Figure 4). This may reflect the use of a CTL line rather than a clone in this experiment, the higher resistance of melanoma cells to CTL lysis, or the lower antigen density of HMW-MAA compared to CD20. However, this experiment still demonstrates that a tumour-specific cell surface marker (unlike CD20) can be used to sensitize tumour cells to lysis by virus-specific CTL by targeting with soluble MHC class I/peptide complexes.

The feasibility of employing such a targeting system in vivo remains to be assessed. A multi-step targeting system may not be necessary, since tumour-specific antibodies could potentially be conjugated to MHC class I/peptide complexes prior to administration. Nevertheless, it is apparent that multi-layer antibody–avidin delivery systems are viable in clinical research (Magnani et al, 1995), with the high affinity ($10^{-15}$ M) non-covalent bond formed between biotin and avidin being exploited in antibody targeting systems to deliver effector mechanisms including radioactive isotopes (Paganelli et al, 1991) and tumour necrosis factor (Moro et al, 1997). The immunogenicity of avidin or other components of the targeting system may limit repeated use; however, such responses might be minimized by transient immunosuppression (Ledermann et al, 1991). Alternative less immunogenic two-step delivery systems, such as the recently described calmodulin–calmodulin binding peptide system (Neri et al, 1996), may also become available clinically.

Regardless of the chemistry used, however, the stability of the MHC class I/peptide complexes at 37°C will need further investigation, as the rate of degradation observed may preclude use in vivo. Although our data show good stability over 20 h, this is unlikely to be sufficient for clinical purposes. Fortunately, it is likely that complex stability will be significantly improved by either protein engineering methods (Toshitani et al, 1996; Lone et al, 1998) or chemical modification (Wilson et al, 1995), and new methods for synthesizing MHC class I/peptide complexes are already being assessed in this regard (unpublished data). Whilst there is no experience to date on the administration of recombinant HLA molecules in humans, it seems likely that they may circulate freely after administration, since endogenous soluble MHC class I molecules are readily detectable in healthy serum (Davies et al, 1989). Further, since monomeric MHC class I/peptide complexes have a low affinity for the T-cell receptor (Schneck et al, 1989; Altman et al, 1996), binding to specific CTL during circulation, which might cause sequestration of complexes in lymphoid areas, is likely to be minimal. Administration of MHC class I/peptide complexes in animal experiments has not proven toxic (Terness et al, 1996). Work is currently in progress to examine the pharmacokinetics of the current system in preclinical models.

The experiments presented here used complexes containing a peptide from the gag protein of the HIV virus, which would not necessarily be ideal for in vivo application. For clinical work, MHC class I molecules refolded around peptides from the Epstein–Barr virus (EBV) may be a more effective choice. In EBV infection, CTL specific for the RAKFFQLL epitope of the lytic protein BZLF1 can account for up to 44% of peripheral blood CD8+ cells in the acute phase. Data suggest the anti-EBV CTL response persists at significant levels for years after primary infection, and may be repeatedly re-activated during life (Callan et al, 1998), providing natural boosts in the frequency and activation of CTL which might be re-targeted at tumours.

Alternatively, novel immunogenic peptides or alloreactive HLA molecules may induce strong effector function. In vivo cytokine support with interleukin-2, which up-regulates T-cell activity, or the infusion of antigen-specific CTL expanded ex vivo are other modifications which may also aid clinical utility.

Finally it must be noted that whilst many tumour types express TAAs, heterogeneity in the level of expression does occur, so some tumour cells may not be targeted by antibody and lysed directly. However, in vitro data from the analogous antibody-superantigen system shows that the high local levels of cytokines released by activated T-cells can lead to the death of untargeted bystander tumour (Dohlsten et al, 1995), it is likely that similar effects will occur in a targeting system using MHC class I/peptide complexes. Similarly, it is possible that the presence of activated CTL releasing cytokines in the tumour may lead to enhancement of a specific anti-tumour immune response.

### REFERENCES

Altman JD, Moss PAH, Goulder PJR, Barouch DH, McHeyzer-Williams MG, Bell JL, McMichael AJ and Davis MM (1996) Phenotypic analysis of antigen-specific T lymphocytes. *Science* **274**: 94–96

Berenson RJ, Bensinger WI, Kalamasz D and Martin P (1986) Elimination of Daudi lymphoblasts from human bone marrow using avidin–biotin immunoadsorption. *Blood* **67**: 509–515

Bodmer WF, Browning MJ, Krausa P, Rowan A, Bicknell DC and Bodmer JG (1993) Tumour escape from immune response by variation in HLA expression and other mechanisms. *Ann NY Acad Sci* **690**: 42–49

Buraggi GL, Callegaro L, Mariani G, Turrin A, Cascinelli N, Attili A, Bombardieri E, Terno G, Plassio G, Dovis M, Mazzuca N, Natali PG, Scassellati GA, Rosa U and Ferrone S (1985) Imaging with $^{131}$I-labeled monoclonal antibodies to a high molecular weight melanoma-associated antigen in patients with melanoma: efficacy of whole immunoglobulin and its $F(ab')_2$ fragments. *Cancer Res* **45**: 3378–3387

Callan MFC, Tan L, Annels N, Ogg GS, Wilson JDK, O'Callaghan CA, Steven N, McMichael AJ and Rickinson AB (1998) Direct visualisation of antigen-specific CD8+ T cells during the primary immune response to Epstein–Barr virus in vivo. *J Exp Med* **187**: 1395–1402

© 2000 Cancer Research Campaign 

Davies HS, Pollard SG and Calne RY (1989) Soluble HLA antigens in the circulation of liver graft recipients. *Transplantation* **47**: 524–527

Dohlsten M, Hedlund G, Akerblom E, Lando PA and Kalland T (1991) Monoclonal antibody-targeted superantigens: a different class of anti-tumour agents. *Proc Natl Acad Sci USA* **88**: 9287–9291

Dohlsten M, Sundstedt A, Bjorkland M, Hedlund G and Kalland T (1993) Superantigen-induced cytokines suppress growth of human colon-carcinoma cells. *Int J Cancer* **54**: 482–488

Dohlsten M, Lando PA, Bjork P, Abrahmsen L, Ohlsson L, Lind P and Kalland T (1995) Immunotherapy of human colon cancer by antibody-targeted superantigens. *Cancer Immunol Immunother* **41**: 162–168

Eisenbarth GS, Haynes BF, Schroer JA and Fauci AS (1980) Production of monoclonal antibodies reacting with peripheral blood mononuclear cell surface differentiation antigens. *J Immunol* **124**: 1237–1244

Elliot TJ and Eisen HN (1988) Allorecognition of purified major histocompatability complex glyco proteins by cytotoxic T lymphocytes. *Proc Natl Acad Sci USA* **85**: 2728–2732

Garboczi DN, Hung DT and Wiley DC (1992) HLA-A2–peptide complexes: refolding and crystallization of molecules expressed in *Escherichia coli* and complexed with single antigenic peptides. *Proc Natl Acad Sci USA* **89**: 3429–3433

Hird V, Maraveyas A, Snook D, Dhokia B, Soutter WP, Meares C, Stewart JS, Mason P, Lambert HE and Epenetos AA (1993) Adjuvant therapy of ovarian cancer with radioactive monoclonal antibody. *Br J Cancer* **68**: 403–406

Kane KP, Sherman LA and Mescher MF (1989) Molecular interactions required for triggering alloantigen-specific cytolytic T lymphocytes. *J Immunol* **142**: 4153–4160

Klein E, Klein G, Nadkarni JS, Nadkarni JJ, Wigzell H and Clifford P (1968) Surface IgM-kappa specificty on a Burkitt lymphoma cell in vivo and in derived cell culture lines. *Cancer Res* **28**: 1300–1310

Knuth A, Wolfel T, Klehmann E, Boon T and Meyer zum Buschenfelde KH (1989) Cytolytic T-cell clones against an autologous human melanoma: specificty study and definition of three antigens by immunoselection. *Proc Natl Acad Sci USA* **86**: 2804–2808

Ledermann JA, Begent RH, Massof C, Kelly AM, Adam T and Bagshawe K (1991) A phase-1 study of repeated therapy with radiolabelled antibody to carcinoembryonic antigen using intermittent or continuous administration of cyclosporin A to suppress the immune response. *Int J Cancer* **47**: 659–664

Lone YC, Motta I, Mottez E, Guilloux Y, Lim A, Demay F, Levraud JP, Kourilsky P and Abastado JP (1998) In vitro induction of specific cytotoxic T lymphocytes using recombinant single-chain MHC class I/peptide complexes. *J Immunother* **21**: 293–294

Magnani P, Paganelli G, Modorati G, Zito F, Songini C, Sudati F, Koch P, Maecke HR, Brancato R, Siccardi AG and Fazio F (1995) Quantitative comparison of direct antibody labeling and tumor pre-targeting in uveal melanoma. *J Nucl Med* **37**: 967–971

Maloney DG, Liles TM, Czerwinski DK, Waldichuk C, Rosenberg J, Grillo-Lopez, A and Levy R (1994) Phase I clinical trial using escalating single-dose infusion of chimeric anti-CD20 monoclonal antibody (IDEC-C2B8) in patients with recurrent B-cell lymphoma. *Blood* **84**: 2457–2466

Moro M, Pelagi M, Fulci G, Paganelli G, Dellabona P, Casorati G, Siccardi AG and Corti A (1997) Tumor cell targeting with antibody–avidin complexes and biotinylated tumor necrosis factor alpha. *Cancer Res* **57**: 1922–1928

Neri D, Natali PG, Petrul H, Soldani P, Nicotra MR, Vola R, Rivella A, Creighton AM, Neri P and Mariani M (1996) Recombinant anti-human melanoma antibodies are versatile molecules. *J Invest Dermatol* **107**: 164–170

Ogg GS, X, Jin S, Bonhoeffer PR, Dunbar MA, Nowak S, Monard JP, Segal Y, Cao SL, Rowland-Jones V, Cerundolo A, Hurley M, Markowitz DD, Ho DF, Nixon and McMichael AJ (1998) Quantitation of HIV-1-specific cytotoxic T lymphocytes and plasma load of viral RNA. *Science* **279**: 2103

Paganelli G, Magnani P, Zito F, Villa E, Sudati F, Lopalco L, Rosetti C et al; (1991) Three-step monoclonal antibody tumor targeting in carcinoembryonic antigen-positive patients. *Cancer Res* **51**: 5960–5966

Parham P, Barnstable C and Bodmer W (1979) Use of a monoclonal antibody (W6/32) in structural studies of HLA-A,B,C antigens. *J Immunol* **123**: 342–349

Parker KC, Bednarek MA, Hull LK, Utz U, Cunningham B, Zweerik HJ, Biddison WE and Coligan JE (1992) Sequence motifs important for peptide binding to the human MHC class I molecule HLA-A2. *J Immunol* **149**: 3580–3587

Perez P, Hoffman RW, Shaw S, Bluestone JA and Segal DM (1985) Specific targeting of cytotoxic T cells by anti-T3 linked to an anti-target cell antibody. *Nature* **316**: 354–356

Riethmuller G and Johnson JP (1992) Monoclonal antibodies in the detection and therapy of micrometastatic epithelial cancers. *Curr Opin Immunol* **4**: 647–655

Riethmuller G, Holz E, Schlimok G, Schmiegel W, Raab R, Hoffken K, Gruber R, Funke I, Pichlmaier H, Hirche H, Buggisch P, Witte J and Pichlmayr R (1998) Monoclonal antibody therapy for resected Dukes' C colorectal cancer: seven-year outcome of a multicenter randomized trial. *J Clin Oncol* **16**: 1788–1794

Romero P, Gervois N, Schneider J,, Escobar P Valmori D, Pannetier C, Steinle A, Wolfel T, Lienard D, Brichard V, van Pel A, Jotereau F, and Cerottini JC (1997) Cytolytic T lymphocyte recognition of the immunodominant HLA-A*0201-restricted Melan-A/MART-1 antigenic peptide in melanoma. *J Immunol* **159**: 2366

Schneck J, Lee Maloy W, Coligan JE and Margulies DH (1989) Inhibition of an allospecific T cell hybridoma by soluble class I proteins and peptides: estimation of the affinity of a T cell receptor for MHC. *Cell* **56**: 47–55

Terness P, Dufter C, Otto G, Opelz G (1996) Allograft survival following immunization with membrane-bound or soluble peptide MHC class I donor antigens: factors relevant for the induction of rejection by indirect recognition. *Transplant Int* **9**: 2–8

Toshitani K, Braud V, Browning MJ, Murray N, McMichael AJ and Bodmer WF (1996) Expression of a single chain HLA class I molecule in a human cell line: presentation of exogenous peptide and processed antigen to cytotoxic T lymphocytes. *Proc Natl Acad Sci USA* **93**: 236–240

Townsend A and Bodmer H (1989) Antigen recognition by class I restricted T lymphocytes. *Annu Rev Immunol* **7**: 601–624

Valone FH, Kaufman PA, Guyre PM, Lewis LD, Memoli V, Deo Y, Graziano R, Fisher JL, Meyer L, Mrozek-Orlowski M, Wardwell K, Guyre V, Morley TL, Arvizu C and Fanger MW (1995) Phase Ia/Ib trial of bispecific antibody MDX-210 in patients with advanced breast or ovarian cancer that overexpresses the proto-oncogene HER-2/neu. *J Clin Oncol* **13**: 2281–2292

Weiner LM, Alpaugh RK, Amoroso AR, Adams GP, Ring DB and Barth MW (1996) Human neutrophil interactions of a bispecific monoclonal antibody targeting tumor and human Fc gamma RIII. *Cancer Immunol Immunother* **42**: 141–150

Wilson JL, Cunningham AC and Kirby JA (1995) Alloantigen presentation by B cells: analysis of the requirement for B cell activation. *Immunology* **86**: 325–333

 *© 2000 Cancer Research Campaign*
